# The Anti-Epileptic Drug Valproic Acid (VPA) Inhibits Steroidogenesis in Bovine Theca and Granulosa Cells *In Vitro*

**DOI:** 10.1371/journal.pone.0049553

**Published:** 2012-11-12

**Authors:** Claire Glister, Leanne Satchell, Anthony E. Michael, Andrew B. Bicknell, Philip G. Knight

**Affiliations:** 1 School of Biological Sciences, University of Reading, Reading, Berkshire, United Kingdom; 2 Division of Biomedical Sciences, St George’s University of London, London, United Kingdom; VU University Medical Center, The Netherlands

## Abstract

Valproic acid (VPA) is used widely to treat epilepsy and bipolar disorder. Women undergoing VPA treatment reportedly have an increased incidence of polycystic ovarian syndrome (PCOS)-like symptoms including hyperandrogenism and oligo- or amenorrhoea. To investigate potential direct effects of VPA on ovarian steroidogenesis we used primary bovine theca (TC) and granulosa (GC) cells maintained under conditions that preserve their ‘follicular’ phenotype. Effects of VPA (7.8–500 µg/ml) on TC were tested with/without LH. Effects of VPA on GC were tested with/without FSH or IGF analogue. VPA reduced (P<0.0001) both basal (70% suppression; IC_50_ 67±10 µg/ml) and LH-induced (93% suppression; IC_50_ 58±10 µg/ml) androstenedione secretion by TC. VPA reduced *CYP17A1* mRNA abundance (>99% decrease; P<0.0001) with lesser effects on *LHR*, *STAR*, *CYP11A1* and *HSD3B1* mRNA (<90% decrease; P<0.05). VPA only reduced TC progesterone secretion induced by the highest (luteinizing) LH dose tested; TC number was unaffected by VPA. At higher concentrations (125–500 µg/ml) VPA inhibited basal, FSH- and IGF-stimulated estradiol secretion (P<0.0001) by GC without affecting progesterone secretion or cell number. VPA reversed FSH-induced upregulation of *CYP19A1* and *HSD17B1* mRNA abundance (P<0.001). The potent histone deacetylase (HDAC) inhibitors trichostatin A and scriptaid also suppressed TC androstenedione secretion and granulosal cell oestrogen secretion suggesting that the action of VPA reflects its HDAC inhibitory properties. In conclusion, these findings refute the hypothesis that VPA has a direct stimulatory action on TC androgen output. On the contrary, VPA inhibits both LH-dependent androgen production and FSH/IGF-dependent estradiol production in this *in vitro* bovine model, likely by inhibition of HDAC.

## Introduction

Epilepsy is a common disorder affecting over 1% of the population, including almost one million women of child bearing age [1]. Therapeutic drugs successfully control seizures in about 70% of patients. However, medication is long-term and side effects are common; these include effects on the reproductive endocrine system of both males and females (reviews: [2], [3], [4]). One of the most widely prescribed anti-epileptic drugs is valproic acid (VPA), a branched-chain fatty acid with anti-convulsant and mood stabilizing properties (review [5]). VPA is also used in the treatment of bipolar disorders, migraines and neuropathic pain. The anti-convulsant and mood stabilizing properties of VPA have been attributed to modulation of voltage-dependent sodium channels, enhancement of GABA inhibitory neurotransmission and/or decreased cerebral glucose metabolism [5].VPA is also known to affect various intracellular signal transduction pathways including MAPK, PKB and PKC-mediated pathways [6], [7] as well as being an inhibitor of type 1 histone deacetylase (HDAC) [8].

Over the past 15 years it has emerged that there is an increased incidence of polycystic ovarian syndrome (PCOS)-like symptoms in epileptic women taking VPA suggesting that the drug can perturb ovarian function and androgen synthesis, possibly as a result of multiple effects on the hypothalamic-pituitary-ovarian axis (reviews: [2], [3], [5], [9]). PCOS is a very common reproductive endocrine disorder affecting 6–8% of women of reproductive age [10], [11]; despite intensive research, its aetiology remains largely unknown. PCOS is usually defined by the presence of hyperandrogenism (in the absence of specific adrenal and/or pituitary disease), oligo- or amenorrhoea and characteristic ‘polycystic’ ovarian morphology as revealed by ultrasonography [11], [12]. However, PCOS is also strongly associated with obesity, insulin resistance and hyperinsulinemia, features of the so-called ‘metabolic syndrome’ [10], [12].

The association between VPA treatment and PCOS-like symptoms was first reported by Isojarvi *et al.* [13] who found that almost 50% of women treated for epilepsy with VPA had amenorrhea, oligomenorrhoea or prolonged menstrual cycles compared with 19% of women taking carbamazepine, another anti-epileptic drug. In a later study Isojarvi *et al.* [14] reported that 64% of women receiving VPA had polycystic ovaries and/or hyperandrogenism. In addition to raised serum androgen levels these women were obese, had high fasting serum insulin levels and low levels of serum insulin-like growth factor-binding protein 1. O’Donovan *et al.* [15] reported that 41% of women taking VPA medication for bipolar disorder exhibited PCOS; more women reported menstrual abnormalities in the VPA group (47%) than in the group not receiving VPA (13%). Other studies [16], [17] have documented higher plasma testosterone and free-androgen levels in women treated with VPA.

Several mechanistic studies, both *in vivo* and *in vitro*, have investigated the link between VPA exposure, PCOS-like symptoms and ovarian hyperandrogenism (review: [4]). Most of these have relied on animal models. For instance, Tauboll *et al.* [18] reported that chronic VPA treatment in rats increased the number of follicular ‘cysts’ and raised total ovarian weight but decreased plasma testosterone concentrations. In contrast, Sveberg Roste *et al.* [19] found that chronic VPA exposure in female rats did not affect serum androgen levels but dramatically reduced serum estrogen levels, thus raising the androgen: estrogen ratio. Ferin *et al.* [20] reported that long-term VPA treatment in normally cycling rhesus monkeys had no effect on androgen levels or ovarian morphology. In an *in vitro* study using propagated human ovarian thecal cells (TC) it was shown that VPA treatment augmented ovarian androgen synthesis and increased transcription of steroidogenic genes [21]. However, Fisseha *et al.* [22] reported an inhibitory effect of VPA on hCG-induced androgen secretion by rat theca-interstitial cells. Using a porcine TC-GC co-culture model, Tauboll *et al.* [23] found that VPA increased basal and LH-stimulated androgen secretion by cells from small follicles but decreased LH-stimulated androgen secretion by cells from medium-size follicles. In addition, they showed that VPA reduced basal and FSH-induced estradiol secretion.

Given these inconsistencies in the literature regarding the effects of VPA on the female reproductive endocrine axis, the aim of the present study was to re-evaluate the potential direct ovarian effects of VPA using well-established bovine TC and GC culture models in which the cells retain a non-luteinized ‘follicular’ phenotype and are highly responsive to LH and FSH, respectively. The cow, like the human, is a mono-ovulatory species and human ovaries share many more similarities with bovine ovaries than they do with rodent or porcine ovaries. As such the bovine ovary is regarded as an excellent model for the human ovary [24], [25].

## Results

### Effect of VPA on Basal and LH-induced Androstenedione and Progesterone Secretion by TC

**Figure 1:**
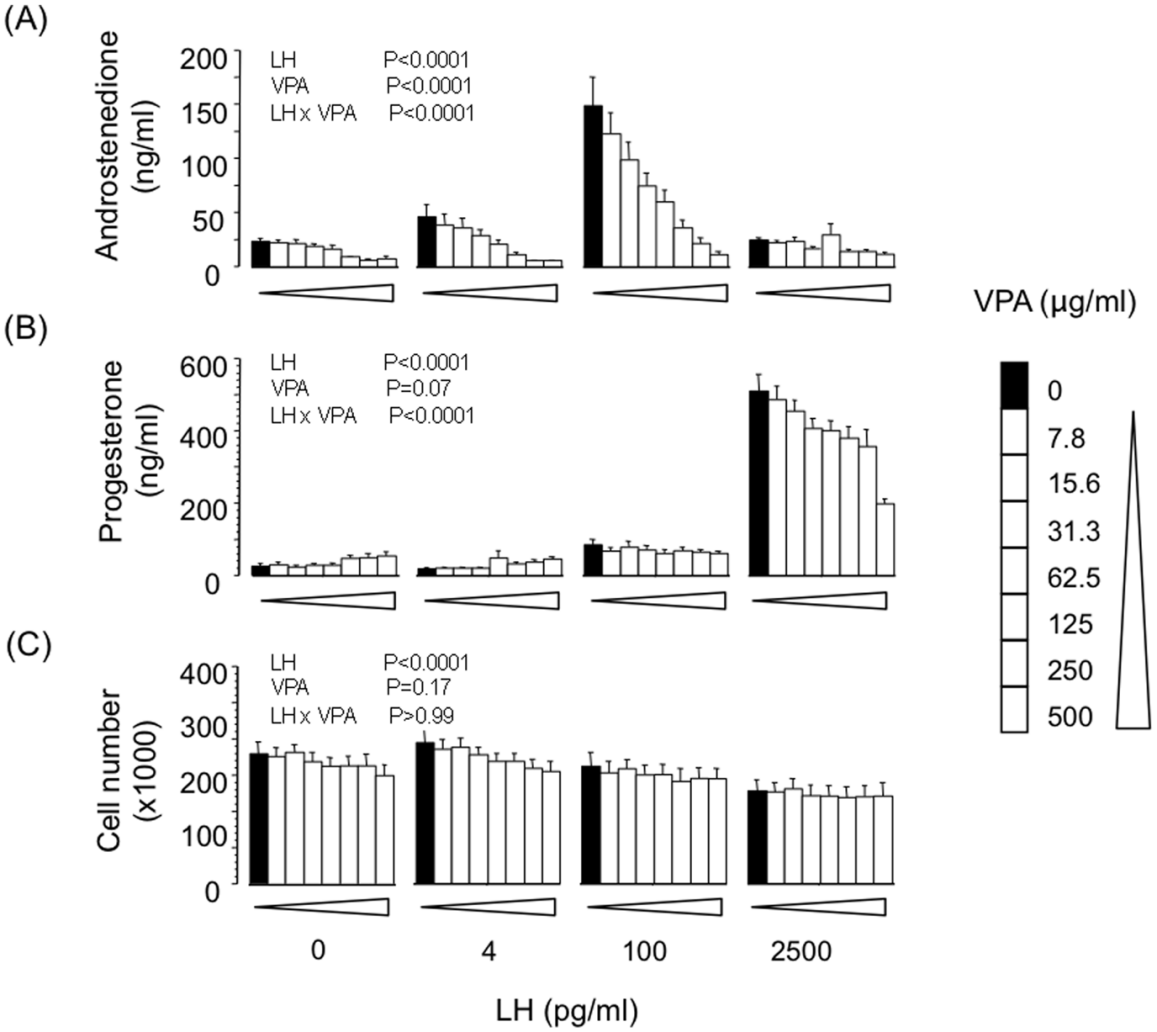
Effects of VPA on basal and LH-induced secretion of (A) androstenedione and (B) progesterone by bovine theca-interna cells; panel (C) shows viable cell number at the end of culture. Values are means ± SEM (n = 4 independent experiments). Results of 2-way ANOVA are indicated.

Consistent with our previous observations using this *in vitro* TC model [26] treatment of cells with an optimal dose-level of LH (100 pg/ml) promoted a robust increase in androstenedione secretion (∼6-fold; P<0.0001) but did not affect progesterone secretion (**Fig. 1**). At a much higher ‘luteinizing’ dose-level (2500 pg/ml) LH promoted a marked increase in secretion of progesterone but not androstenedione. A small though significant (P<0.0001) decrease in cell number was also elicited by LH. VPA had a marked and dose-dependent suppressive effect on basal androstenedione secretion (IC_50_ 67±10 µg/ml; P<0.0001) and androstenedione secretion induced by the optimal dose-level of LH (IC_50_ 58±10 µg/ml; P<0.0001). VPA also had a modest suppressive effect on progesterone secretion (IC_50_>250 µg/ml) induced by the highest LH dose-level. VPA had no effect on viable cell number at the end of culture (**Fig. 1**).

### Effect of VPA and Basal and LH-induced Gene Expression in TC

**Figure 2:**
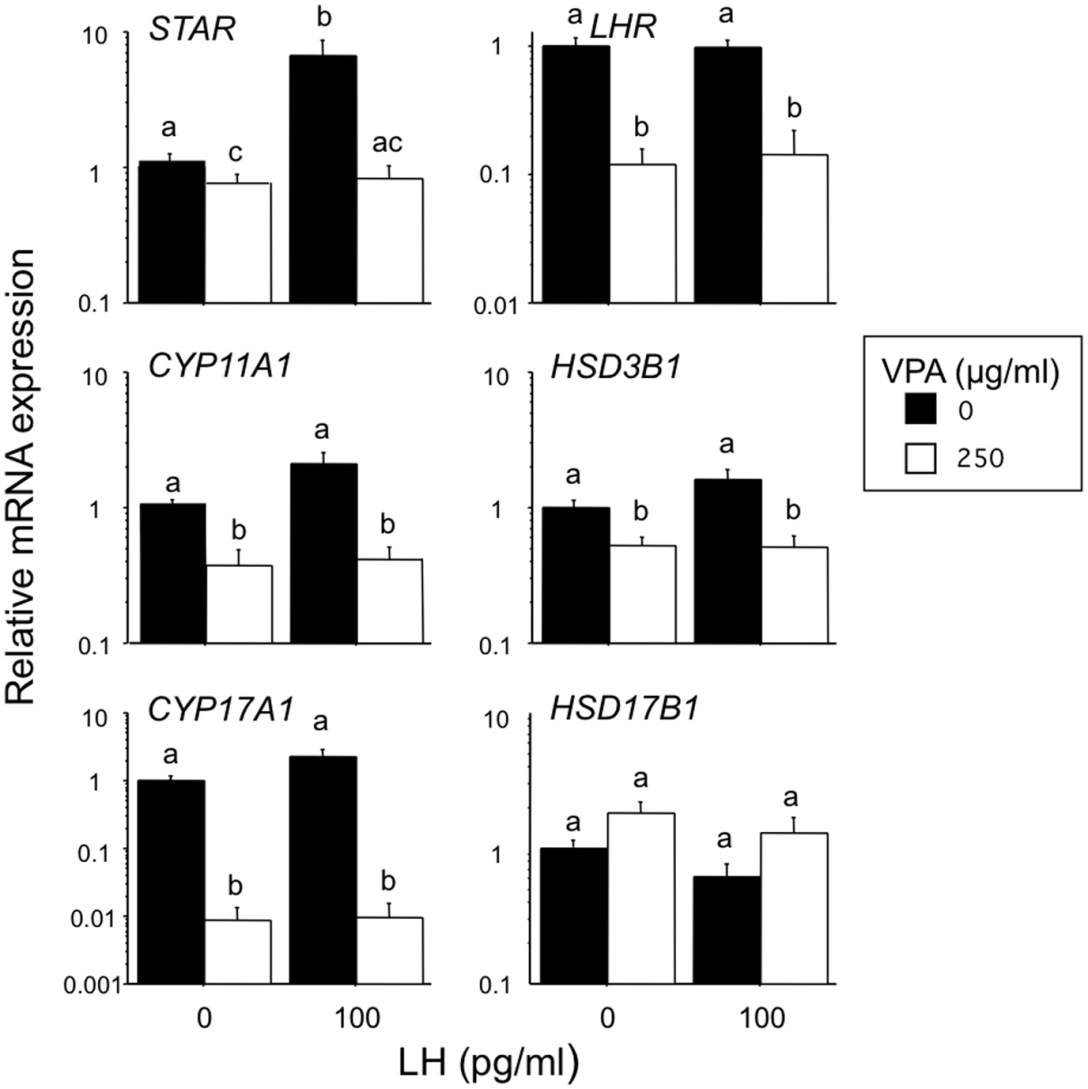
Effects of VPA in the presence and absence of LH on the relative abundance of six key steroidogenic transcripts in bovine theca-interna cells. Values are means ± SEM (n = 7 independent experiments). Means without a common letter are significantly different (P<0.05).

The effects of VPA (250 µg/ml) and LH (100 pg/ml) on the relative abundance of six key mRNA transcripts involved in TC steroidogenesis are shown in **Fig. 2**. LH increased the abundance of *STAR* mRNA (5-fold; P<0.01) and tended to increase *CYP17A1* mRNA (3-fold, P>0.05) but did not significantly affect *LHR*, *CYP11A1*, *HSD3B1* or *HSD17B1* mRNA abundance. VPA had a profound suppressive effect on *CYP17A1* transcript abundance, both in the presence and absence of LH stimulation (>99% reduction; P<0.0001). VPA also reduced expression of *LHR*, *STAR*, *CYP11A1* and *HSD3B1* under basal and LH-stimulated conditions (∼50% to 90% reduction; P<0.05) but to a much lesser extent than the suppression of *CYP17A1* mRNA.

### Effect of VPA on Basal, FSH- and IGF-induced Estradiol and Progesterone Secretion by GC

**Figure 3:**
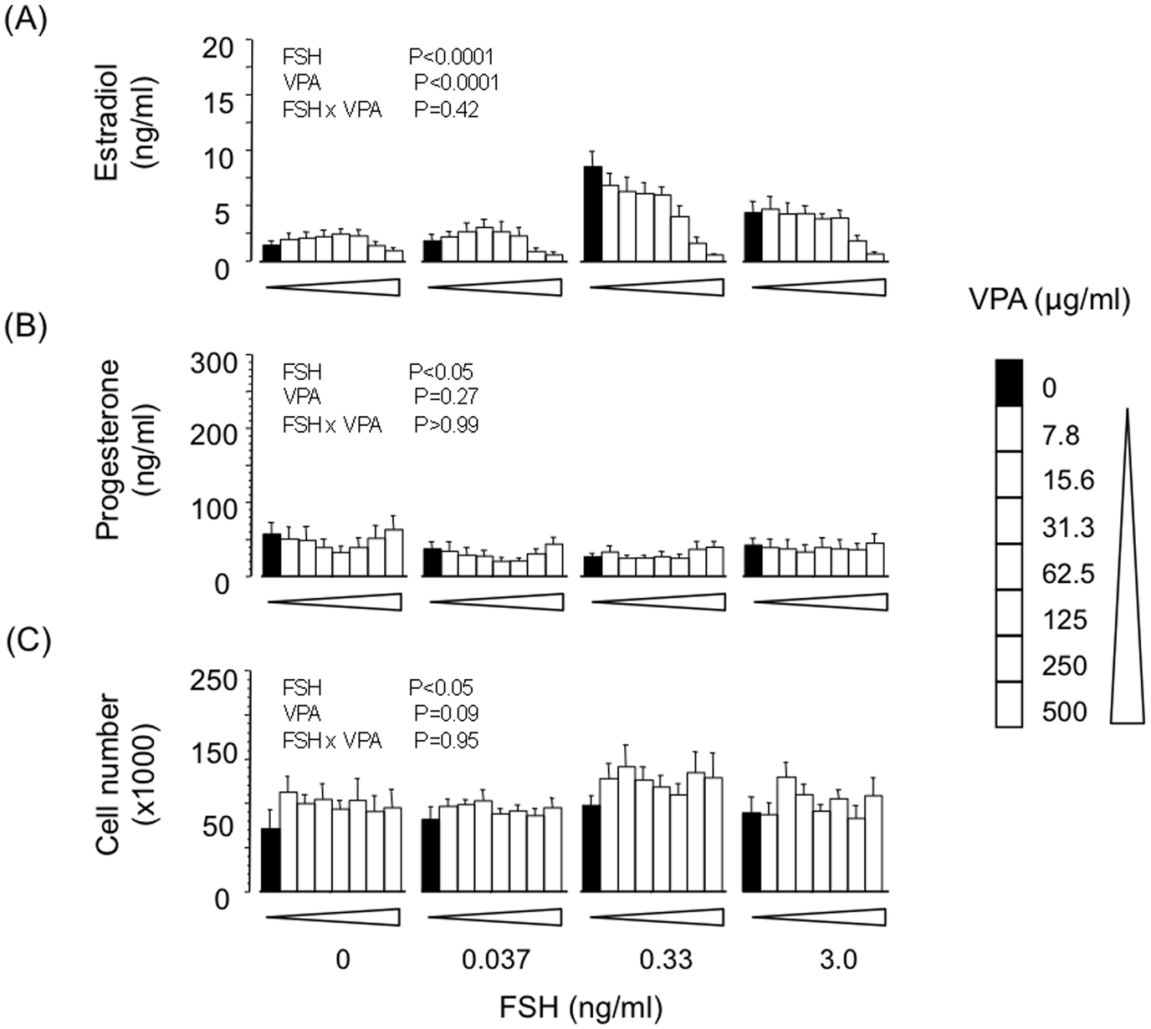
Effects of VPA on basal and FSH-induced secretion of (A) estradiol and (B) progesterone by bovine granulosa cells; panel (C) shows viable cell number at the end of culture. Values are means ± SEM (n = 4 independent experiments). Results of 2-way ANOVA are indicated.

**Figure 4:**
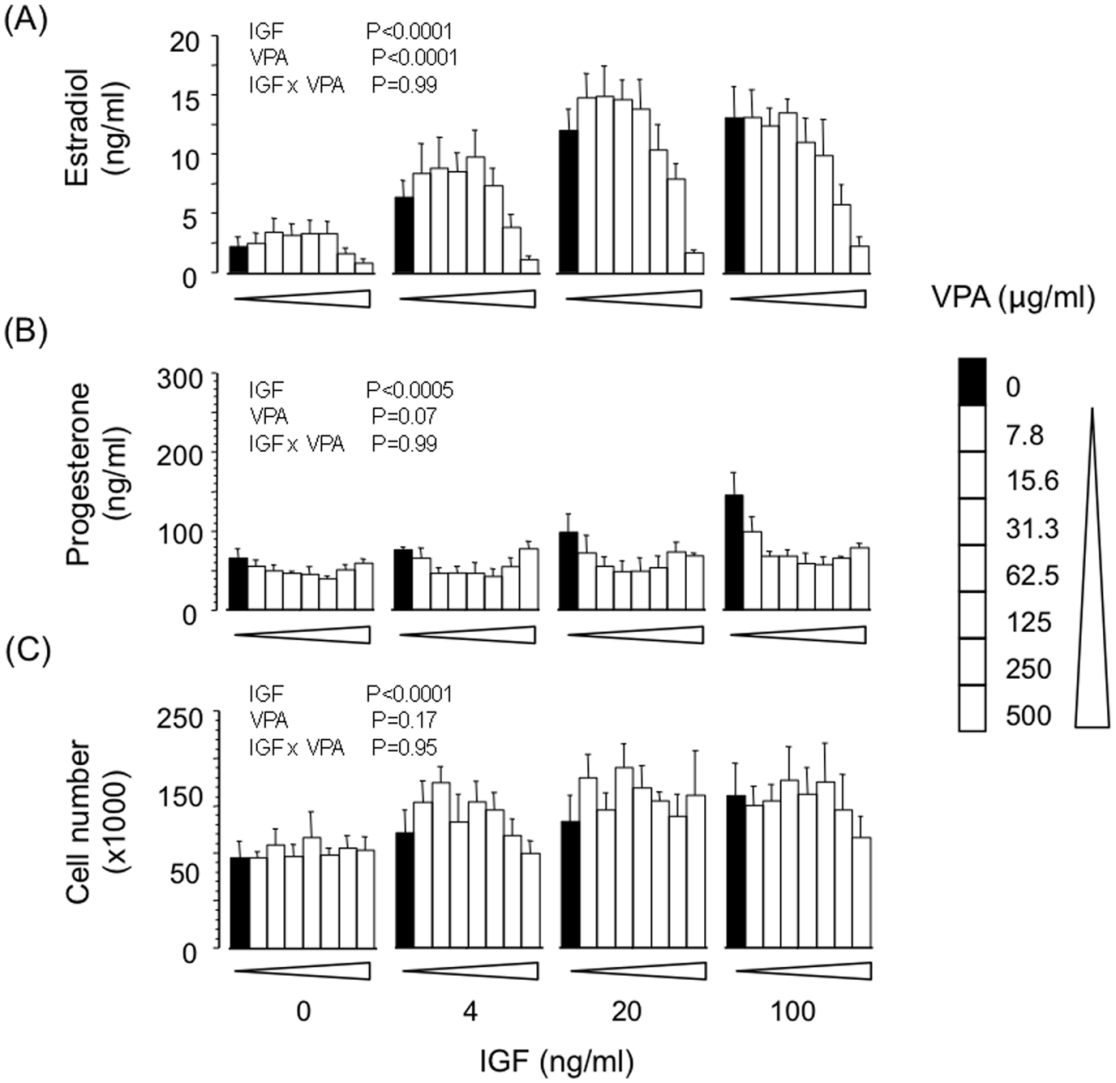
Effects of VPA on basal and LR3-IGF-1 (IGF)-induced secretion of (A) estradiol and (B) progesterone by bovine granulosa cells; panel (C) shows viable cell number at the end of culture. Values are means ± SEM (n = 4 independent experiments). Results of 2-way ANOVA are indicated.

VPA dose-dependently suppressed basal (IC_50_ ∼250 µg/ml; P<0.01) and FSH-induced (IC_50_ ∼200 µg/ml; P<0.0001) secretion of estradiol but did not influence progesterone secretion or viable cell number at the end of culture (**Fig. 3**). Similarly, VPA dose-dependently reversed the stimulatory effect of IGF analogue on estradiol secretion (IC_50_ ∼250 µg/ml; P<0.001) without affecting progesterone secretion or cell number (**Fig. 4**).

### Effect of VPA on Basal, FSH-induced and IGF-induced Gene Expression in GC

**Figure 5:**
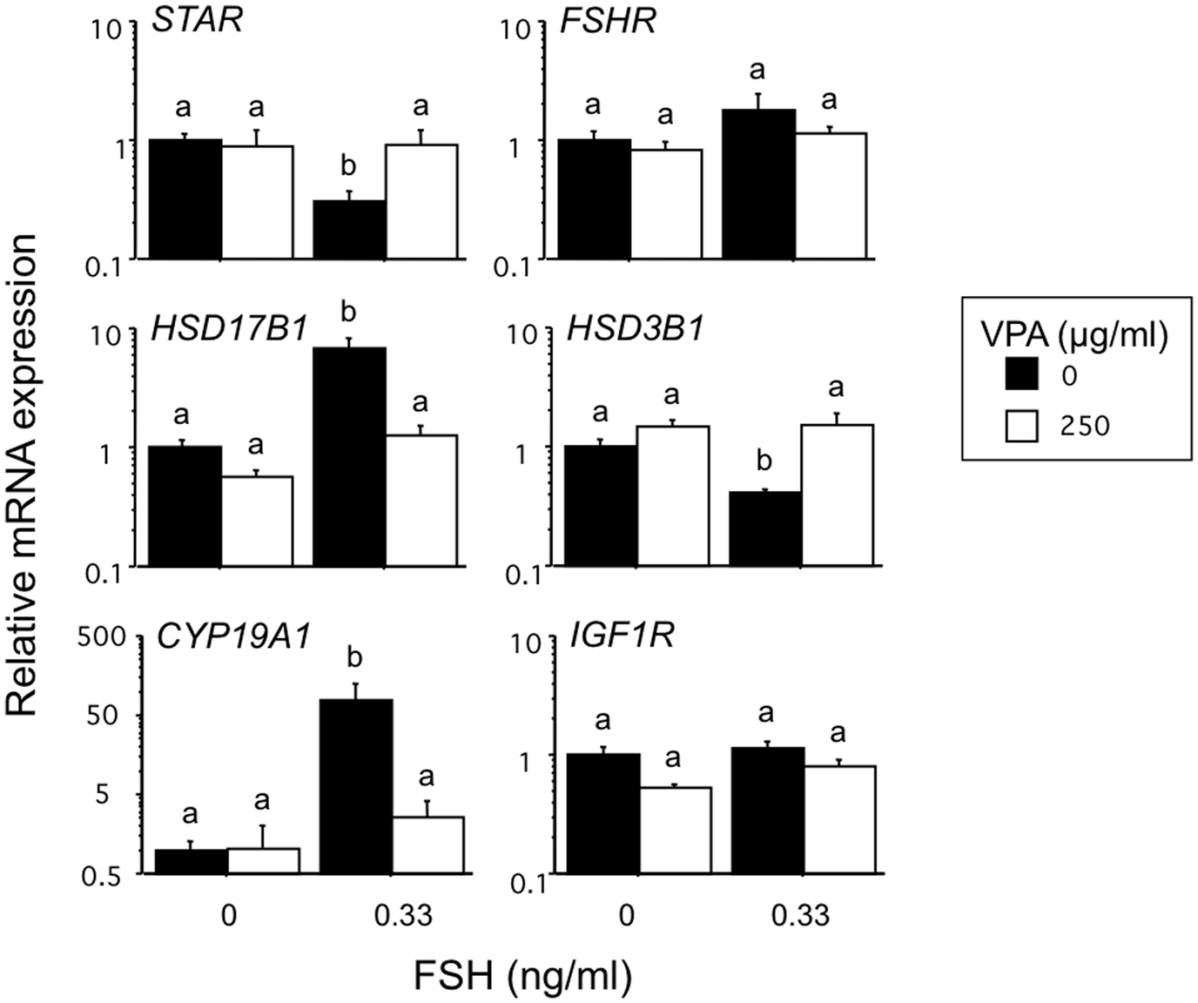
Effects of VPA in the presence and absence of FSH on the relative abundance of six key steroidogenic transcripts in bovine granulosa cells. Values are means ± SEM (n = 4 independent experiments). Means without a common letter are significantly different (P<0.05).

As anticipated FSH greatly increased the relative abundance of *CYP19A1* (76-fold; P<0.0001) and *HSD17B1* (7-fold; P<0.01) mRNA in GC, paralleling the FSH-induced increase in estradiol secretion by the cells (**Fig. 5**). Concomitantly, FSH slightly reduced the abundance of *STAR* and *HSD3B1* mRNA, consistent with the small reduction in progesterone secretion observed. VPA reversed the FSH-induced upregulation of *CYP19A1* and *HSD17B1* expression (P<0.001). Likewise the FSH-induced reduction in *STAR* and *HSD3B1* expression was reversed by VPA treatment. Neither FSH or VPA, alone or in combination, had any significant effect on abundance of FSHR or IGF1R transcripts.

**Figure 6:**
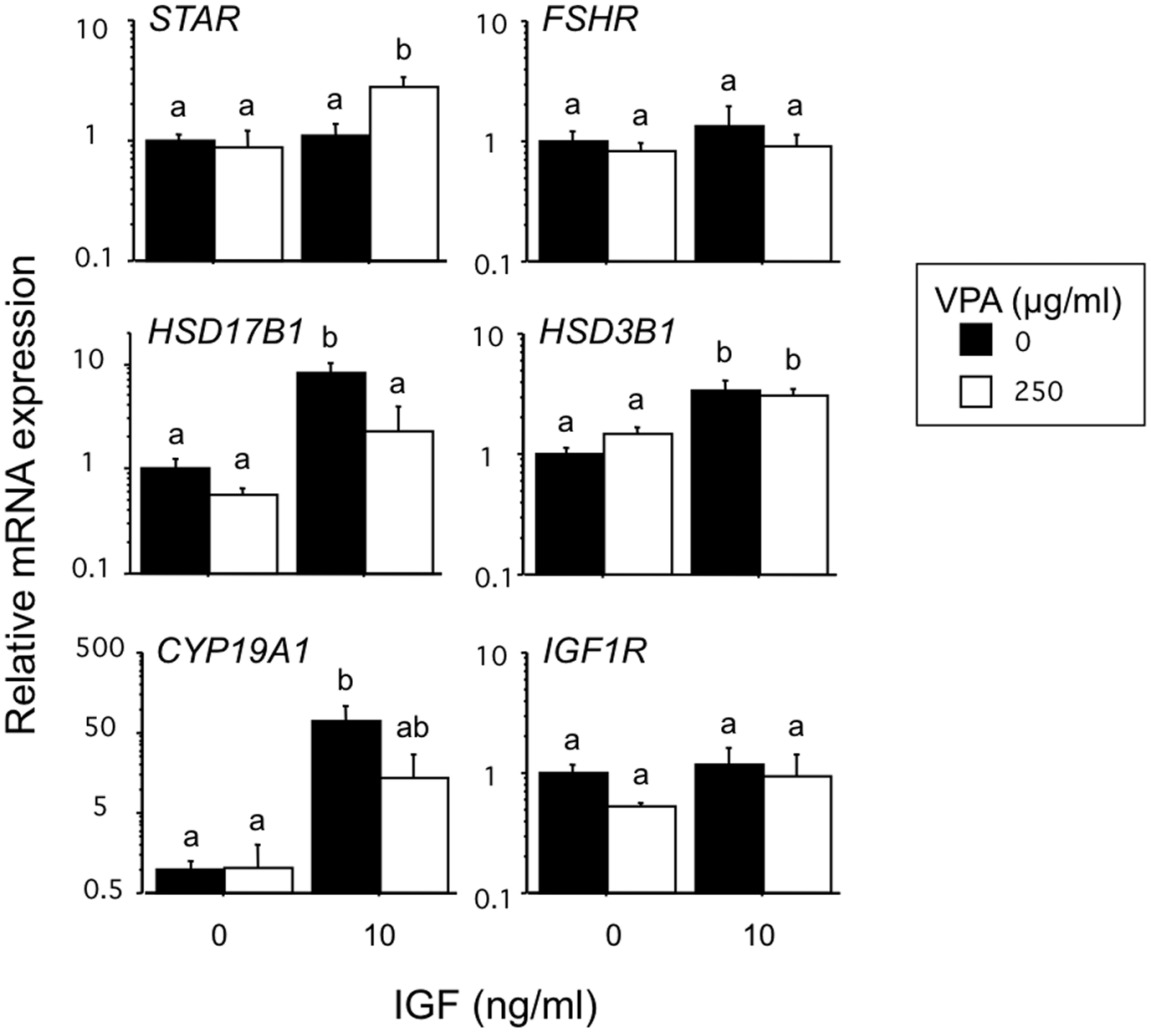
Effects of VPA in the presence and absence of LR3-IGF on the relative abundance of six key steroidogenic transcripts in bovine granulosa cells. Values are means ± SEM (n = 4 independent experiments). Means without a common letter are significantly different (P<0.05).

As shown in **Fig. 6** treatment of GC with IGF analogue also upregulated the abundance of CYP19A1 (71-fold; P<0.0001) and HSD17B1 (8-fold; P<0.01) transcripts consistent with the observed IGF-induced increase in estradiol secretion. Co-treatment with VPA partially reversed these increases in CYP19A1 and HSD17B1 mRNA. Neither IGF analogue or VPA, alone or in combination, had any significant effect on abundance of FSHR or IGF1R transcripts in GC.

### Comparison of the Effect of Three Different HDAC Inhibitors on TC Androgen Secretion and GC Estrogen Secretion

**Figure 7:**
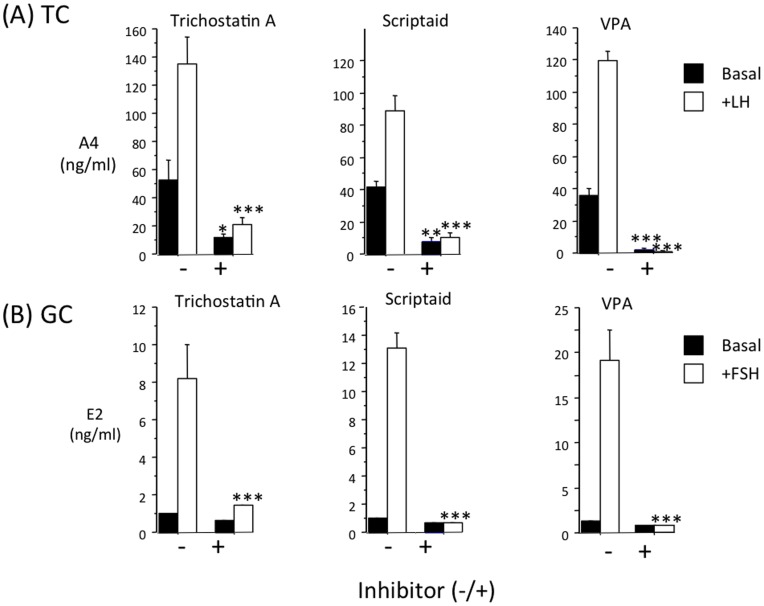
Three different HDAC inhibitors (trichostatin A; 5 nM), scriptaid; 500 nM) and VPA; 2 mM) have similar inhibitory effects on (A) basal and LH-induced androstenedione secretion by bovine theca cells and (B) basal and FSH-induced estrogen secretion by bovine granulosa cells. Values are means ± SEM (n = 4 independent cultures). *p<0.05, ***p<0.001 compared to corresponding group without inhibitor.

To explore the possibility that the suppressive action of VPA on TC androgen secretion and GC estrogen secretion reflects its HDAC inhibitory properties, we conducted a further experiment to compare the effects of VPA (250 µg/ml; ∼2 mM) with those of two highly potent HDAC inhibitors, scriptaid and trichostatin A at 5 nM and 500 nM, respectively. As shown in Fig. 7 all three compounds inhibited basal and LH-induced secretion of androstenedione by TC and FSH-induced secretion of estrogen by GC.

**Table 1:** List of primers used for real-time PCR.

Target	Accessionnumber	Forward primer5' to 3'	Reverse primer5' to 3'	Ampliconsize (bp)
*FSHR*	NM_174061.1	GCCAGCCTCACCTACCCCAGC	AATTGGATGAAGGTCAGAGGTTTGCC	75
*LHR*	NM_174381.1	ATTGCCTCAGTCGATGCCCAGACC	AAAAAGCCAGCCGCGCTGC	92
*IGF1R*	XM_606794.3	ACCTCCCAGCCTAAGCAAAATGATCC	TCTTCGGCCACCATGCAGTTCC	123
*STAR*	NM_174189	TTTTTTCCTGGGTCCTGACAGCGTC	ACAACCTGATCCTTGGGTTCTGCACC	103
*CYP11A1*	NM_176644	CAGTGTCCCTCTGCTCAACGTCC	TTATTGAAAATTGTGTCCCATGCGG	99
*HSD3B1*	NM_174343.2	GCCACCTAGTGACTCTTTCCAACAGCG	TGGTTTTCTGCTTGGCTTCCTCCC	111
*CYP17A1*	NM_174304	GACAAAGGCACAGACGTTGTGGTCA	TGATCTGCAAGACGAGACTGGCATG	301
*CYP19A1*	NM_174305.1	CGCCACTGAGTTGATTTTTGCTGAGA	TAAGGCTTTGCGCATGACCAGGTC	301
*HSD17B1*	NM_001102365	CGCATATTGGTGACCGGGAGCATA	AATCGCCAGACTCTCGCACAAACC	108
*ACTB*	NM_173979.3	ATCACCATCGGCAATGAGCGGTTC	CGGATGTCGACGTCACACTTCATGA	128

## Discussion

In view of considerable evidence that women undergoing VPA treatment for epilepsy and bipolar disorder show an increased incidence of PCOS-like symptoms including hyperandrogenemia and menstrual cycle disturbances (reviewed by: [2], [3], [5]), research is clearly warranted to try to clarify the mechanism(s) through which this widely prescribed drug might elicit such an effect on ovarian function. Potentially VPA could perturb ovarian function by acting at one or more levels of the hypothalamic-pituitary-ovarian axis, or by modifying some other organ that influences ovarian function indirectly (e.g. pancreatic insulin secretion). This study, together with several previous studies discussed below, sought evidence for a direct action of VPA on ovarian steroidogenesis. Importantly, the present *in vitro* data presented firmly refute the hypothesis that VPA has a direct stimulatory effect on thecal androgen production.

Using well defined in vitro model systems based on primary cultures of bovine TC [26], [27], [28], [29] and GC [30], [31], [32], [33] the present study shows that VPA has a direct, dose-dependent inhibitory action on ovarian steroidogenesis, suppressing both basal and LH-induced androgen production by TC as well as FSH- and IGF-induced estrogen production by GC. With regard to TC, our findings concur with those of a recent study [22] documenting a VPA-induced inhibition of basal and hCG-induced androgen secretion by rat theca-interstitial cells. Moreover, chronic VPA treatment *in vivo* was shown to decrease plasma testosterone concentrations in rats [18]. However, these findings contradict those of a study on propagated (4^th^ passage) human TC [21] showing that VPA stimulated basal and forskolin-induced androgen secretion, and raised cellular levels of CYP17A1 (cytochrome P450c17) and CYP11A1 (cytochrome P450scc) protein. In another *in vitro* study utilizing a porcine TC/GC co-culture model [23] VPA was found to stimulate androgen secretion by small follicles but to inhibit androgen secretion by medium-size follicles. The reasons for these inconsistencies amongst *in vitro* studies in different laboratories are currently unknown but species differences are likely a major contributory factor, along with methodological differences with regard to culture conditions (cell harvesting, plating density, culture media, use of serum supplementation, culture duration, extent to which cells have been propagated) that may affect the behaviour and/or phenotype of cells *in vitro*. In the case of the porcine TC/GC co-culture model, another factor complicating the interpretation of these data is the ability of porcine TC to synthesize considerable amounts of estrogen [34]. Bovine TC, like those of human, mouse, rat and sheep, do not express *CYP19A1* and are incapable of estrogen synthesis.

The short term primary cultures of bovine TC and GC used in the present study are believed to provide a physiologically relevant model in the sense that the cells retain a non-luteinized (‘follicular’) phenotype over the 6-day culture period under chemically defined, serum-free conditions. These cells are highly responsive to gonadotrophin stimulation in terms of LH-induced androgen secretion by TC, FSH- and IGF-induced estradiol, inhibin and activin secretion by GC and expression of genes involved in steroidogenesis including *LHR* and *CYP17A1* in TC and *FSHR* and *CYP19A1* in GC [26], [27], [28], [29], [31], [32], [33], [35], [36].

Examination of steady-state mRNA levels in control and VPA-treated TC revealed a clear-cut inhibition by VPA of five key genes involved in the steroidogenic response, most notably *CYP17A1* (>99% suppression) with a much lesser degree of inhibition of *LHR*, *STAR*, *CYP11A1* and *HSD3B1* transcript abundance. Likewise, Fisseha et al. [22] showed that VPA reduced *CYP17A1* expression by rat theca-interstitial cells cultured under both basal and hCG-stimulated conditions, although effects on *LHR*, *STAR*, *CYP11A1* and *HSD3B1* expression were not reported. These authors also showed a comparable inhibitory effect of VPA on the androgen response to 8-bromo-cyclic adenosine-3′5′-monophosphate (8-Br-cAMP), indicating that VPA exerts its suppressive action at a level distal to cAMP generation following LH/CG receptor activation.

The profound suppression of both basal and LH-induced androgen secretion we observed can best be accounted for by the severe reduction in *CYP17A1* expression since progesterone secretion was not suppressed by VPA, except in the presence of a very high dose-level of LH that, as anticipated, promoted cell luteinisation as evidenced by a marked increase in progesterone secretion and a loss of androgen secretion [26], [37]. Indeed, VPA tended to increase progesterone secretion in cells incubated without LH or with a very low concentration of LH (4 pg/ml), indicating that steps in the steroidogenic pathway proximal to CYP17A1 catalysis were not rate-limiting under these conditions. The lack of effect of VPA on viable cell number recorded at the end of culture indicates that VPA does not affect cell proliferation and/or survival during the 4-day treatment period.

With regard to GC, our finding that VPA also suppressed basal, FSH-induced and IGF-induced secretion of estradiol-17β by cultured GC is consistent with several *in vitro* and *in vivo* studies. Using GC harvested from women undergoing laparotomy procedures for infertility treatment Tauboll *et al.* [38] recently reported that VPA suppressed basal and FSH-induced estradiol secretion. Similarly, in the porcine TC/GC co-culture study of Tauboll *et al.* [23] VPA reduced basal and FSH-induced estrogen secretion. Chronic *in vivo* exposure of rats to VPA greatly reduced circulating estrogen levels, although androgen levels were not affected [19]. However, long-term *in vivo* treatment of rhesus monkeys with VPA had no effect on circulating estradiol or androgen levels and did not affect menstrual cyclicity or ovarian morphology [20].

QPCR analysis of GC levels of six key transcripts involved in steroidogenesis revealed that VPA did not affect the abundance of *FSHR* or *IGF1R* mRNA suggesting that the inhibitory action of VPA on estrogen secretion did not involve downregulation of either receptor. However, VPA completely reversed FSH-induced expression of *CYP19A1* and *HSD17B1* and partially reversed IGF-induced *CYP19A1* and *HSD17B1* expression. An inhibitory effect of VPA on *CYP19A1* in FSH-treated human GC was also observed by Tauboll *et al.* [38]. As discussed below the enzymes encoded by *CYP19A1* and *HSD17B1* both play key roles in the synthesis of estradiol-17β by GC.

VPA was some 4-fold more potent in suppressing TC androgen production (IC_50_ ∼60 µg/ml) than GC estradiol production (IC_50_ ∼250 µg/ml), the former value being within the therapeutic serum range for human subjects of about 50–100 µg/ml [39]. Since TC-derived androgens are an obligatory substrate for GC the CYP19A1 (cytochrome P450 aromatase) enzyme, and hence follicular estrogen synthesis [40], [41], in an *in vivo* context this implies a dual inhibitory action of VPA on follicular estrogen production primarily by depriving GC of aromatase substrate and secondarily by inhibiting FSH- and IGF-induced upregulation of *CYP19A1* and *HSD17B1* expression by GC. Androstenedione is the principle androgen produced by bovine (and human) TC, and the17β-hydroxysteroid dehydrogenase enzyme (HSD17B1) is required to convert the product of its aromatization, estrone, into estradiol-17β, the principle ovarian estrogen [40], [41]. Despite the above, on the basis of the relative IC_50_ values, it is more likely that the predominant direct ovarian effect of VPA at therapeutically relevant concentrations is exerted on TC rather than GC.

The extent to which observations made using different animal models, including the present bovine ovarian cell culture systems, can be extrapolated to human ovarian function is uncertain. However, in contrast to rodent and porcine ovaries used for many of the previous VPA-associated studies, there are striking similarities between human and bovine ovaries in terms of morphology, follicle size-range, developmental time-line, steroidogenic activity, ovulation rate and physiological regulation by endocrine and intraovarian factors [24], [25], [42], [43]. Given these similarities, and considering the practical and ethical constraints associated with accessing human ovarian tissue for *in vitro* studies, the likelihood is that insights gained from pharmacological studies using bovine *in vitro* models are indeed relevant to translational and biomedical research on human ovarian function.

With regard to the mechanism through which VPA suppresses androgen secretion by theca cells, our observation that two highly potent HDAC inhibitors, scriptaid (pan-HDAC inhibitor) and trichostatin A (type 1and 2 HDAC inhibitor) also suppressed androstenedione secretion, supports the notion that the action of VPA is due to its activity as a type 1 HDAC inhibitor [8], [44], [45]. Since TC expression of LH receptor and each of the steroidogenic proteins suppressed by VPA (STAR, CYP11A1, CYP17A1 and HSD3B1) is known to be partially dependent on the common transcription factor, steroidogenic factor-1 (SF-1; NR5A1) [46], we speculate that repressed transcription and/or posttranslational modification of SF-1 could provide a plausible explanation for the inhibition of androgen synthesis observed under both basal and LH-stimulated conditions. In support of this, a previous study using steroidogenic adrenocortical cell lines found that VPA (and other HDAC inhibitors) targets SF-1 for ubiquitination and degradation [47]. Clearly, further mechanistic studies are required to delineate the cellular and molecular pathways through which VPA and other HDAC inhibitors modulate ovarian steroidogenesis at the level of both theca and granulosa cells. The observation that all three HDAC inhibitors also inhibited FSH-induced estradiol secretion by GC suggests a similar mechanism of action on this cell type.

In conclusion, the present findings do not support the hypothesis that VPA has a direct action on the ovary to raise thecal androgen production. Conversely, our evidence firmly indicates a VPA-induced inhibition of both TC androgen production and GC estrogen production arising primarily from a suppression of *CYP17A1* and *CYP19A1* mRNA levels in the respective cell-types. Irrespective of the mechanism of action of VPA, our findings underscore the need for further in vivo studies, particularly clinical studies in humans, to re-evaluate whether this widely prescribed drug does indeed promote hyperandrogenism through a direct stimulatory action on ovarian theca cells.

## Materials and Methods

### Bovine Ovaries and Cell Culture

Bovine GC and TC were isolated from adult bovine ovaries obtained from a local abattoir (St Merryn Meat Ltd) as described in detail elsewhere [26], [27], [32]. For each experiment cells pooled from approximately 50 individual 4–6 mm follicles were routinely plated out in 96-well tissue culture plates (Nunclon, Life Technologies Ltd, Paisley, UK) at 75,000 viable cells/well (estimated using trypan blue) and cultured for 6 days under defined serum-free conditions. The culture medium used for both cell-types consisted of McCoy’s 5A modified medium supplemented with 1% (v/v) antibiotic-antimycotic solution, 10 ng/ml bovine insulin, 2 mM L-glutamine, 10 mM HEPES, 5 µg/ml apo-transferrin, 5 ng/ml sodium selenite and 0.1% (w/v) BSA (all purchased from Sigma). In the case of GC the culture medium was supplemented with androstenedione (Sigma UK Ltd, Poole, Dorset, UK) at 10^−7 ^mol/l as a substrate for CYP19A1 (cytochrome P450 aromatase). Medium was removed after 48 h and 96 h and replaced with fresh media containing treatments as indicated below. Conditioned media were retained for assay and at the end of the culture period viable cell number was determined by neutral red dye uptake assay to provide an assessment of cell proliferation/survival.

In culture experiments in which total RNA was to be extracted for PCR analysis, cells were seeded into 24-well plates (10^5^ cells/ml) with 3 replicate wells per treatment. At the end of culture cell lysates were prepared using Tri-Reagent (Sigma) and pooled lysates from replicate wells were stored at −80C until total RNA isolation.

### Treatments

Ovine LH (NIADDK oLH-S-16) and FSH (NIADDK oFSH-19SIAPP) were obtained from NHPP, Torrance, CA, USA. Recombinant IGF-1 analogue (Long R3 IGF-1) and VPA, were purchased from Sigma. Treatments were dissolved in Hank’s balanced salt solution containing 0.1% (w/v) BSA and stock solutions sterilized using 0.2 µm membrane filters before dilution in sterile culture medium. Scriptaid (Sigma) and trichostatin A (Sigma) were dissolved in DMSO (10 mM) before further dilution in sterile culture medium.

### Steroid Assays

Concentrations of androstenedione in TC-conditioned media were determined by radioimmunoassay as reported previously [26]. The detection limit was 0.1 ng/ml and mean intra- and inter-assay CVs were 7% and 10% respectively. Concentrations of estradiol-17β in GC-conditioned media were determined by radioimmunoassay [48]. The detection limit of the assay was 2 pg/ml and mean intra- and inter-assay CVs were 6% and 9% respectively. Concentrations of progesterone in conditioned media from both cell-types were determined by competitive ELISA [49]. The detection limit was 0.1 ng/ml and mean intra- and inter-assay CVs were 8% and 11% respectively.

### Purification of RNA, cDNA Synthesis and Real-time PCR

Total RNA was isolated from cultured cells using Tri-Reagent (Sigma). Briefly, cell monolayers were lysed in Tri Reagent (0.5 ml/well) and after aqueous phase separation, RNA was precipitated in isopropanol, washed in 75% (v/v) ethanol and the RNA pellet re-suspended in 50 µl nuclease-free water. Potential genomic DNA contamination was removed with an RNase-free DNase kit (RQ1; Promega UK Ltd, Southampton, UK). The Tri Reagent extraction process was repeated and the final RNA pellet re-suspended in 20 µl nuclease-free water; RNA quantity and quality were evaluated by spectrophotometry at 260/280 nm. First strand cDNA was synthesized from 1 µg of RNA template using the Reverse-iT reverse transcription kit (used according to manufacturers protocol; Abgene, Epsom, Surrey, UK) in a 20 µl reaction primed with random hexamers.

Primers (see **table 1**) were designed to amplify target sequences using Primer Express software (version 1.5, Applied Biosystems). In primer validation experiments melt curve analysis and agarose gel electrophoresis were used to verify that each primer pair generated a single product of the predicted size. cDNA template log-dilution curves were used to demonstrate satisfactory PCR efficiency (>85%) and linearity. PCR assays were carried out in a volume of 24 µl, comprising 10 µl cDNA template (equivalent to 20 ng reverse-transcribed RNA), 1µl each forward and reverse primers (final concentration 0.4 µM) and 12 µl QuantiTect SYBR Green QPCR 2× Master Mix (Qiagen, UK). Samples were processed on an ABI PRISM® 7700 Sequence Detection System (Perkin Elmer-Applied Biosystems, Warrington, UK) with the following thermal cycling conditions: 2 min at 50°C, 15 min at 95°C (one cycle) followed by 15 s at 95°C and 1 min at 60°C (40 cycles).

The ΔΔCt method [50] was used for comparison of the relative abundance of each mRNA transcript in TC and GC. Ct values for each transcript in a given sample were first normalized to β-actin Ct value (which was uniform across experimental all groups: ANOVA P>0.1). Resultant ΔCt values for individual replicates within each treatment group were then normalized to the average ΔCt value of the respective vehicle-treated control group. These ΔΔCt values were finally converted to fold-differences using the formula: fold-difference = 2^(−ΔΔCt)^.

### Statistical Analysis

Results for hormone secretion (during final 96–144 h period of culture) and cell number at the end of culture were analysed using two-way analysis of variance (ANOVA) and are presented as means ± SEM based on 4 independent culture experiments. To reduce heterogeneity of variance, hormone data were log-transformed prior to statistical analysis. QPCR data (from n = 7 independent TC batches, n = 4 independent GC batches) were statistically analysed (ANOVA and *post-hoc* Fisher’s PLSD test) as ΔCt values before conversion to fold-difference values for graphical presentation.
